# Socio-ecological determinants of older people’s mental health and well-being during COVID-19: A qualitative analysis within the Irish context

**DOI:** 10.3389/fpubh.2023.1148758

**Published:** 2023-03-23

**Authors:** Viveka Guzman, Frank Doyle, Ronan Foley, Peter Craven, Noelene Crowe, Penny Wilson, Ailbhe Smith, Gerry Hegarty, Maria M. Pertl

**Affiliations:** ^1^Department of Health Psychology, School of Population Health, Royal College of Surgeons in Ireland, Dublin, Ireland; ^2^Department of Geography, Maynooth University, Maynooth, Ireland; ^3^Well-being, Interventions and Support During Epidemics (WISE) Study Research Advisory Group, Royal College of Surgeons in Ireland, Dublin, Ireland

**Keywords:** aging population, COVID-19 pandemic, socio-ecological determinants, mental health, well-being, qualitative research

## Abstract

**Background:**

Evidence indicates that older people with biological and social vulnerabilities are at high risk of short- and long-term consequences related to the COVID-19 pandemic. However, studies have also highlighted that the crisis may present opportunities for personal growth if older individuals are met with appropriate resources and support.

**Objective:**

The aim of this study was to explore the perceptions of older people regarding how individual, social, and environmental factors have supported or hindered their well-being and health during COVID-19.

**Methods:**

We analyzed data collected between April–May and October–November 2021 from the Well-being, Interventions and Support during Epidemics (WISE) study, a qualitative investigation of community-dwellers based in Ireland and aged 65 years or over. Participants (*n* = 57) completed written submissions, narrative interviews and/or go-along interviews detailing their experiences during the pandemic. Framework analysis was carried out in NVivo 12 to identify determinants, linkages, and explanations within Bronfenbrenner’s socio-ecological model.

**Results:**

The mean age of participants was 74.9 years, 53% were female, 45% lived alone, and 86% lived in areas with high urban influence. Our findings highlight the heterogeneous effect of COVID-19 across diverse older individuals who held distinct concerns, capabilities, and roles in society before and during the pandemic. Multi-scalar contextual characteristics such as individual’s living arrangements, neighborhood social and built environments, as well as social expectations about aging and help seeking, had an influential role in participants’ well-being and available supports. We identified mixed views regarding public health restrictions, but a consensus emerged questioning the suitability of one-size-fits-all approaches based on chronological age.

**Conclusions:**

Our results suggest that some negative pandemic consequences could have been avoided by increasing collaboration with older people and with the provision of clearer communications. The interdependencies identified between individual characteristics and socio-ecological factors that influenced participants’ availability of supports and development of adaptive strategies represent areas of opportunity for the development of age-friendly interventions during and beyond public health crises.

## 1. Introduction

The highest proportion of hospitalizations in intensive care units and mortality rates during the COVID-19 pandemic have been observed amongst older populations (1–3). The health risk is unequally distributed and the poorest prognoses have been observed among older individuals who experience both biological and social vulnerabilities (4). The influence of these risk factors is not limited to the prevention and course of a COVID-19 infection, but significantly contributes to broader economic and social consequences that may affect older people’s quality of life and well-being in the short- and long-term. A deterioration in older people’s mental health and well-being during the pandemic may also be compounded by previous social isolation and loneliness, increased sedentary behaviors and limited access to healthcare services for non-COVID needs (5–7). Additionally, age-specific public health measures, such as exclusive hours to carry on essential activities and sheltering-in-place (also known as cocooning), have created among older individuals ambivalent emotions of feeling protected and feeling ostracized (8). However, emerging research also indicates that some older individuals have experienced positive changes and enhanced meaning of life during the pandemic (9, 10).

An in-depth understanding of the barriers and enablers to well-being experienced by older people during the pandemic is needed to identify characteristics associated with resilient and vulnerable individuals, and to develop appropriate support interventions. Given the heterogeneity of potential factors associated with older people’s mental health and well-being in the pandemic context, a socio-ecological perspective is best suited for consideration of diverse social, material and affective determinants embedded at multiple levels of influence (11–13). A growing body of studies utilizing quantitative approaches has provided valuable insights into some of the predictors associated with mental health and well-being outcomes related to COVID-19. However, most of these studies have explored only one level of influence or type of determinant. For instance, evidence has emerged from studies focused on psychological and socio-demographic characteristics (14, 15), health behaviors (16), social resources (17, 18), and access to nature and/or outdoor environments (19, 20). However, limited attention has been given to possible interactions between multiple levels and/or possible intersections within determinants. Moreover, the variables utilized within existing analyses can rely on researchers’ preconceived assumptions and experiences of other public challenges that may differ from COVID-19.

Findings from qualitative studies provide a more nuanced portrayal of older people’s experiences in diverse contexts, and additional insights into the complexity inherent in health-related outcomes during the pandemic. For instance, findings on how older adults based in Switzerland made sense of the pandemic during the first lockdown suggest several levels of influence may produce ambivalent affects in the same individual (8). Similarly, a study based in Northern Texas identified some individual, social and environmental factors that supported early resilience in marginalized older adults (21). However, little attention has been given to how these factors interact with each other according to those who experience them. Additionally, to date most of the existing qualitative evidence pertains to the first months of the pandemic, so it remains unclear how determinants at multiple levels shape adaptation strategies in the longer-term.

To fill this gap, the aim of our study is to explore the experiences of those aging-in-place after 1 year of the pandemic onset in Ireland, and to identify enabling and hindering health and well-being determinants across the multiple levels of influence proposed by Bronfenbrenner’s socio-ecological model. Levels of analysis include individual factors, the immediate environment of everyday life (micro-system), interactions between diverse everyday spheres (meso-system), broader environments where the individual may not be directly involved (exo-system), shared socio-cultural norms, values and ideologies (macro-system), and changes occurring through time (chrono-system) (11). This theoretical framework allows us to contextualize older people’s experiences, and to deepen our understanding of the interactions between actors, networks and agencies that contribute to health and well-being during times of a public health crisis. Our exploratory qualitative approach provides the opportunity to focus on the processes underlying the associations between diverse determinants from the participant’s perspectives. Findings from our study contribute to the evidence of what needs to be done, and for whom, in order to support the health and well-being of individuals aging-in-place during times of social upheaval and massive infectious outbreaks.

## 2. Methodology

### 2.1. Study design

The data analyzed are part of the WISE study, for which a detailed protocol has been published (22). Briefly, utilizing a convenient sampling approach, people aged 65 years and over who were living in community settings across Ireland, were invited to share their experiences during the COVID-19 pandemic through a narrative interview, a written submission, and/or a go-along interview. Participants completed a brief background questionnaire of socio-demographic characteristics (23). Written submissions were open-ended, and researchers provided a few prompts that participants could choose to use to reflect on their experiences (23). Narrative interviews were conducted over the phone or by videoconferencing software, and followed a topic guide touching upon their experiences, perceived stressors, supports available and concerns for the future (23). Go-along interviews utilized prompts to gain a deeper understanding of participants’ lived experiences at a location chosen by them to showcase places of meaning during the pandemic (23).

From the conceptualization of the study, we explored our positionality as researchers and the potential impact of our own experiences, assumptions, and biases in the data collection, analysis, and interpretation of data. These discussions were recorded in our research log and allowed us to consider our insider/outsider perspectives and to adapt our methods accordingly. All participants provided written informed consent before participation.

### 2.2. Study context

The data analyzed was collected between April and early-May 2021 (narrative interviews and written submissions), and between October to November 2021 (go-along interviews). At the first point of data collection, Ireland was experiencing Wave 3 of the pandemic and had confirmed a total number of 223,142 cases with a mortality rate of 96.6 per 100,000 population (24). During early stages of the pandemic in Ireland, public health advice emphasized hygienic measures, such as respiratory etiquette and appropriate hand-washing, and wider initiatives included a prohibition of gatherings and a mandate to stay within a 2 km radius from home (24). For people over 70 years or those extremely medically vulnerable, a specific public health measure termed ‘cocooning’ advised people to strictly remain at home and minimize all face-to-face interactions with others (25). With a decrease in the number of new COVID-19 cases, a phased easing of restrictions allowed movements within a 5 km and then 20 km radius from home, reopening of some services and amenities, and outdoor gatherings for a limited number of individuals (26). From mid-August 2020, an increase in the number of cases lead to Wave 2 and prompted the reintroduction of public health restrictions and development of the 5-level plan to live with COVID-19 (24). Leading up to the Christmas holidays, many of the restrictions had been lifted and Ireland saw its worst surge in cases, which led to Wave 3 and the re-introduction of nationwide restrictions. Moreover, by the end of December 2020 the COVID-19 vaccination roll-out for vulnerable and older individuals began (25). From mid-May 2021 onwards there was a wide lifting of restrictions on travel, personal services, retail, outdoor socializing and religious services, which was as a result of satisfactory developments in the number of cases and escalation of vaccination efforts (27).

### 2.3. Public and patient involvement (PPI)

A research advisory panel conformed of five individuals aging-in-place in Irish communities contributed to the study design and development at multiple stages of the research cycle. A detailed account of their contributions according to the Guidance for Reporting Involvement of Patients and the Public- GRIPP2 (28) is available (23).

### 2.4. Analysis

The current analysis comprises accounts from 57 participants who completed a narrative interview (*n* = 44) and/or written submissions (*n* = 17) and/or a go-along interview (*n* = 5). We selected framework analysis as our analytical method due to its suitability to manage a relatively large amount of qualitative data, and the opportunity to explore both *a priori* and emerging issues (29–31). NVivo 12 software was used to organize the data, assist the coding, and track our analytic decisions. We followed the five framework analysis stages outlined by Ritchie and Spencer (32): (1) The first author transcribed audio-recordings and handwritten submissions, imported and organized files in NVivo, and became familiarized with all transcripts and field notes; (2) The full material from the WISE study was categorized in relation to each of the overall research questions (22). A preliminary codebook from analysis of the first 15 interview transcripts was developed to identify determinants at each socio-ecological level and to generate initial codes. Text was included in more than one code if relevant. A second researcher reviewed the coding structure for consistency and completeness; (3) The codebook was iteratively refined through group discussions and codes consolidated into broader categories which were used to systematically analyze the remaining transcripts; (4) A framework matrix was developed by creating a summary of each participant’s experience and perspectives of relevant determinants at multiple levels; and (5) We compared within and between cases and explored patterns in the data. Determinants’ categories were finalized based on identified relationships between codes and the experiences described by participants. Steps taken to enhance methodological rigor are further detailed in Table 1 according to the Four-Dimensions Criteria (FDC) (33).

**Table 1:** Strategies adopted to establish methodological rigor according to the FDC (33).

Rigor criteria	Study’s strategies
Credibility	Data collection instruments were co-developed in collaboration with 5 experts by experience. Data collection instruments were pilot tested (2 narrative interviews; 1 go-along; 1 written submission). Lead researcher and co-researchers completed training in qualitative study design, analysis, and interpretation. The overall study was supervised by established researchers with expertise in qualitative research. All participants’ data and fieldnotes were stored in a safe location. Data was uploaded and organized with NVivo software.
Dependability	Developed and published a research protocol (22). Kept a detailed research log as a track record of the data collection process and key analytical decisions. Framework matrix constitutes an audit trail of the codes identified, selection of determinants, participants’ summaries, and participants’ quotes.
Confirmability	Completed field notes of preliminary thoughts and interpretations immediately after data collection. Triangulation between written, narrative, and visual data sources, as well as theoretical background on socio-ecological models in general and aging populations.
Transferability	Sample size was guided by principles of information power (34). A multi-method data collection approach was used to facilitate participation opportunities for people with diverse socio-demographic backgrounds, needs & preferences.

## 3. Findings

**Figure 1:**
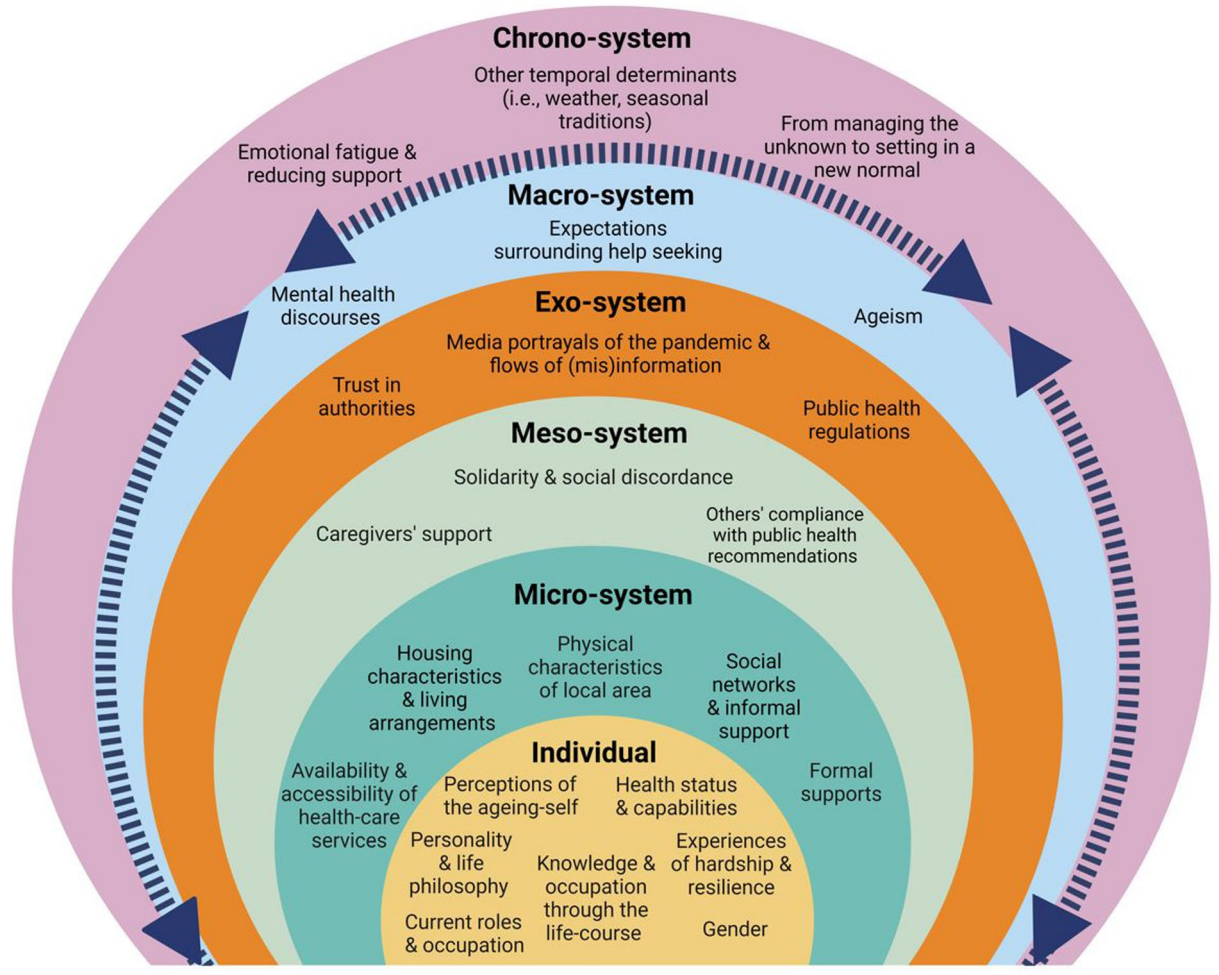
Socio-ecological determinants identified at each level.

The mean age of participants was 74.9 (range 65–96), 53% were female, 45% lived alone, and 86% lived in areas with high urban influence (35). Our analysis identified multiple barriers and enablers that were associated with participants’ health and well-being through diverse levels of the socio-ecological model. Figure 1 provides an overview of the determinants identified at each level, while narrative and tabular representations below provide additional details and quote examples. Participants’ names have been changed for pseudonyms and are followed by their gender (F = female; M = male; NB = non-binary) and age at time of data collection.

### 3.1. Individual level (L1)

The determinants identified at this level (shown in Table 2 with representative quotes) highlight that COVID-19 did not have an equal effect across the older population, but instead it differently affected diverse older individuals who had distinct concerns, capabilities, and roles in society before and during the pandemic. Therefore, individual characteristics such as health status, previous experiences of hardship and personal disposition, played a significant role in enabling or hindering coping mechanisms and adaptive strategies during different stages of the pandemic.

**Table 2:** Details of socio-ecological determinants at the individual level (L1).

Determinant	Definition	Illustrative quotes
1.1- Previous experiences of hardship and resilience	Life-course experiences that have allowed the participant to establish successful coping mechanisms and develop resilience.	“A lot of my life changed about 30 years ago, and I had to take a long journey, which was very, very difficult. So, when this hit, I thought, ‘Oh, I can, I can cope with this, I’ve coped with lots of other things’, and I have coped with it.” Elisa (F, 73 y)
1.2- Personality, character disposition and life philosophy	Personal disposition and interests that influence daily life (i.e., extrovert/introvert, optimistic/negative life outlook, hobbies).	“You cannot change how you deal with life on a day of crisis, you are going to fall back into your normal way of being. So, it’s good to develop a normal way of being that allows you to survive things, and to survive challenges and difficulties and deal with them. Make a plan on a good day so that on a bad day you can fall back on it.” Aoife (F, 66 y)
1.3- Knowledge, roles, and occupation through the life-course	Knowledge acquired during the life-course that provides useful information and skills to cope with the pandemic (i.e., health literacy, digital competency).	“I used to be radiographer and I worked in a hospital. So, I understand the whole transmission of infection stuff and from the very beginning I was very particular about wearing a mask.” Kathleen (F, 68 y)
1.4- Current roles and/or occupation	Current roles and/or occupation- including roles that may support current purpose of life and self-esteem, and/or roles that may be related to enhanced burden during the pandemic (i.e., caregiving for partner or grandchildren, volunteering positions, etc).	“I’m so busy with work things that there’s very little to miss. I’m happy once I’m doing something that’s got a purpose […] I was able to keep volunteering because it was all done by email and phone.” Orla (F, 71 y)
1.5- Perceptions of the aging-self	Personal beliefs about aging and perceptions of oneself as an older person, including comparisons between personal and other’s experiences.	**“**I totally agreed with the spirit of the law, but not the letter of it. It really made me feel that I was 70, and I’m a fit 70-year-old (…) I just felt a bit uncomfortable being lumped with the 90-year-olds and 80-year-olds… I thought the best thing is not to get resentful, just make it work for me.” Siobhan (F, 76 y)
1.6- Gender	Perceptions of the influence of gender in response to stressors and availability of supports during the pandemic.	“Men do not talk face to face. They very much talk shoulder to shoulder when they are doing things, sort of breaks down barriers. With the Men Sheds closed I’d say a lot of men like me found it very tough.” Seamus (M, 76 y)
1.7- Health status and individual capabilities	Perceived impact of medical conditions on personal function and capabilities during the pandemic, including disease symptoms and functional limitations.	“I’m probably a little bit paranoid about catching it. Because I reckon if I caught it, I probably would not survive. I mean because I also have high blood pressure, which I’m taking medication for, you know. So, I definitely think it could be fatal for me, especially, if I got it. So, I have been a bit paranoid about not going anywhere really.” Ruairi (M, 72 y)
1.8- Income and personal finances	Perceptions on the impact of the pandemic on personal economy and capability to support oneself financially.	“We are retired. So, we have enough, you know, our pensions are adequate to live on. We’re not short, we do not have any difficulties like that, which a lot of people have (…) That’s a huge difference that you do not have that kind of concern.” Greg (M, 72 y)

Significantly, individual determinants, such as gender, also interacted with social-expectations and influenced the types of community supports available. For instance, Seamus (see Table 2) referred to the impact of losing face-to-face activities particularly for men who may bond and support each other within physical encounters. While Enda (NB, 66 y), shared that as a gender nonconforming older adult they had to navigate available social networks and cultural expectations during the pandemic:

“I don't have a hetero normative support structure available to me during the pandemic or as I grow older because I didn’t marry, and I don’t have children […] I look for allies where I can find them, and I have what I call my rainbow family. “

Additionally, participants such as Eithne (F, 73 y) highlighted how their perceptions of themselves as aging and/or vulnerable had shifted due to the emphasis on chronological age during the pandemic:

“I keep trying to do everything, keep doing things as I had been doing, but that the pandemic put an end to that really, because it just made the older years seem very real. So, now I can only do what I'm allowed to do health wise and with the general rules that are imposed on you from outside.”

As in the case of Eithne, other participants also reported that the pandemic had forced them to view themselves as old and vulnerable, even if this was not their self-perception beforehand. This resulted from the combination of assumptions about the older population among the general population, as well as the public messaging regarding the restrictions and the behaviors of others that were shaped by the pandemic circumstances across the following socio-ecological levels.

### 3.2. Micro-system level (L2)

The influential factors identified at the micro-level (shown in Table 3 with example quotes) include physical characteristics and social interactions within participants’ proximate environments that enabled or hindered the fulfilment of basic needs and opportunities to take part in valued activities. Features of residential places that allowed interactions with others at a safe physical distance, facilitated exercise and/or provided contact with the outdoors, such as porches and gardens, were mentioned as beneficial for mental and physical health. Views from home to pleasant landscapes including nature, wildlife or other people were also supportive of positive emotions and “good mood.” However, one of the most relevant determinants was the fit between the home characteristics and individual needs and capabilities, which was illustrated by Odhran (NB, 66 y):

**Table 3:** Detailed socio-ecological determinants at the micro-system (L2).

Determinant	Definition	Illustrative quotes
2.1- Housing characteristics and living arrangements	Characteristics of participants’ dwelling environments and living arrangements that enable or hinder their health and wellbeing during the pandemic.	“The hardest part for me was keeping my family out, and the grandchildren (…) But in all fairness, like, you know, we are Travellers and family is very important… and it’s just the living conditions with Travellers as well. I mean, I live in group housing, but there are some of the Travellers living in halting sites and there’re very bad conditions, they may not have running hot water, and the right facilities.” Nan (F, 75 y)
2.2-Physical characteristics of local area- including natural and built environments	Neighborhood characteristics that enable or hinder health and wellbeing during the pandemic (i.e., population density, walkability, accessibility to desirable places such as shops or places with nature).	“This lockdown is tough because the 5k is very tough. If you could travel within your county at least you’ll be able to go to different woods or different forest or different lake, you know, and it’s amazing how just going to a different forest or a different lake cheers you up because you are seeing new trees, new grass, new whatever**”** Tara (F, 66 y)
2.3- Availability and accessibility of health-care services/support	Opportunities and barriers to access adequate health-care services for COVID-19 and non-COVID-19 needs in a timely manner.	**“**I need care that is not available to me at the moment because the HSE^a^ is crushing under the weight of COVID-19 and all. You know, my cancer treatment has been suspended, cancelled. It’s clearly not working for me, and I’m having to settle for less and less and less, you know, lowering the bar of expectation. I have no expectation of survival, and the irony is, I do not think it’s gonna be COVID that’s gonna kill me.” Kevin (NB, 66 y)
2.4- Social networks and informal support from family members, friends, and neighbors	Characteristics of social networks and their role to support and facilitate access to resources to satisfy basic and higher order needs.	“During the COVID-19 Pandemic our children have been very supportive right throughout with visits, phone calls, texts and a constant supply of home-cooked meals. I do not know what we do without our children. They have been the biggest help and reassured me that my wife and I must have done something right.” Paul (M, 86 y)
2.5- Formal non-medical supports from community organizations and the government	Characteristics of formal supports from local authorities, volunteering, and community organizations (i.e., support lines, access to home repairs, access/delivery of food, medication, and other necessary goods, etc.)	“There was a bit of relief that we were being looked after. There were a lot of things being but in place. Like if you had problems, you know, if you cannot get your shopping, you phone up this phone at the local guards or something like that. So, a feeling that yes, we were being protected, and there was the possibility of being looked after, rather than simply shut away. That was a big positive.” Grainne (F, 74 y)

a The Health Service Executive (HSE) is the publicly funded healthcare system in Ireland, responsible for the provision of health and personal social services.

“The housing that I'm in is inadequate. I mean, I’m in a flat all by myself, I have a beautiful view of the Irish Sea which I'm grateful for, but I need home health care. I don't think it's going to arrive in time quite frankly, and I'm alone here you know, which is quite dangerous. I can't really climb the stairs any longer. I'm a prisoner here.”

Public health measures also led to participants spending more time in local areas that saw an increase in “more people cycling, more people walking, more people just being aware of what’s in their own neighborhood” (Niamh, F, 71 y). However, access to enabling neighborhood characteristics varied across participants and conflict sometimes arose between users who had different views of public health measures. For instance, Thomas (M, 72 y) described how his health conditions made proximate environments unsuitable for him to engage in physical activity:

“Where I live, just on the other side of the bridge into [*anonymized location*] it's very hilly. I have no problem walking down the hill but with heart failure, I have a problem walking back up. So, I had to take the car to go to somewhere that was flat in order to walk. So that's what I did, I did exceed the five kilometers I'm afraid.”

Participants also referred to newfound advantages and disadvantages of urban and rural living. For instance, participants based in rural settings mentioned it had been easier to maintain physical distance since their homes were in low-density areas. However, they also mentioned that local services, shops, and amenities were often located beyond the catchment areas allowed by public health measures which curtailed their sense of independence, as in Steven’s (M, 72 y) case:

“The town we usually go to is maybe 25 kilometers away. I just can't go and do stuff that I would have done, you know, very, very simple things […] I have felt down occasionally, I'm probably a bit more emotional about things, and it's probably something to do with being kind of locked up.”

Remote living environments also increased social isolation in participants who did not drive and who relied heavily on remote contact through the phone, post, and digital technologies. In this sense, close relationships, either in terms of geographical proximity (i.e., neighbors) or kinship provided significant emotional support. For instance, Gerald (M, 72 y) reported:

“Just being able to chat with people that you really love and respect and care for, it's very positive and it allows you to look beyond the immediate situation and to realize how incredibly lucky we are here.”

Additionally, formal, and informal social networks helped to fulfil basic and higher order needs, which was detailed by Sean (M, 72 y):

“The things that helped me get through are, number one, the support of friends and neighbors to do shopping, to provide meals and also to offer transport for appointments […] Then, I remember An Post [*Irish postal service*] provided free postcards. I got a number of those sent from friends who couldn't make it here, you know, when we were cocooning. Then contact from the group called social prescribing, I valued their phone calls and the packet of goodies that they sent on two different occasions. Then online support, people were offering to do shopping, like. Also, I have a cat and the local animal welfare group were offering to come and take the cat if I needed to take the cat to the vet. Even the guard [*police*] was available to collect medication from the pharmacy if I couldn't go.”

### 3.3. Meso-system level (L3)

Definitions of the determinants identified at this level and quote examples are provided in Table 4. The overlap between social interactions and the characteristics of public spaces, such as shops or parks, highlights the influence of perceived social solidarity, discordance, and the compliance of others with recommendations. Participants reported they often felt little control in spaces shared with other people, particularly when strangers disregarded the restrictions and/or showcased behaviors associated with increased risk of infection, which led to feelings of stress, anger, anxiety, and fear. In a wider sense, these interactions threatened participants’ sense of community as they felt others did not share the social responsibility of shortening the course of the pandemic or did not care if they carried the disease to other people. Moreover, some participants reported their feelings of dread in public spaces coupled with security resources being deployed elsewhere which led to decreases in foot traffic in certain areas with rising neighborhood insecurity and “groups of dangerous people roaming around” (Deirdre, F, 81 y).

**Table 4:** Details of socio-ecological determinants at the meso-system (L3).

Determinant	Definition	Illustrative quotes
3.1- Solidarity, social discordance, and compliance of others with public health recommendations	Sense of others’ compliance with public health regulations and safety in public spaces.	“A lot of people have been taking shortcuts or having parties and things like that. Unless you all pull in the same direction, you will not achieve the result that you really want to achieve, as soon as you would like to achieve it.” Richard (M, 96 y)
3.2- Support for those providing support	Perceived availability and accessibility of supports for individuals or groups supporting the older person (i.e., family, community groups).	“My daughter, who is a nurse, she is still waiting to get her vaccine, so I’m not happy with that. That’s important because she’s the only one that goes out, and that I have any direct contact with.” Eamon (M, 85 y)

Several participants also noted their own health and wellbeing was facilitated by the opportunity of family, friends and/or caregivers to be supported either by formal or informal interventions, such as the pandemic unemployment payment, availability of PPE for home-visitations, and vaccination roll-out.

### 3.4. Exo-system level (L4)

Definitions of exo-system determinants and quote examples are provided in Table 5. Participants’ narratives indicate a wide spectrum of outlooks concerning the government response to the pandemic with some consensus around the notions that the government “took it seriously” (Ruairi, M, 72 y), and that quick action was needed with limited information. However, several participants questioned the one-size fits all approach based on chronological age and suggested that some unintended consequences could have been avoided by consulting older people’s voices and the provision of clearer communications.

**Table 5:** Detailed socio-ecological determinants at the exo-system (L4).

Determinant	Definition	Illustrative quotes
4.1- Public health regulations for the general and older populations	Positive and negative implications of the implementation of public health regulations to contain the spread of the virus, and specific measures for older people.	“The ironic thing is that the older people are not the vectors, so it is only logical to ask why we are all being locked up… Perhaps one size does not fit all? The blanket approach that has meant that people who are already isolated by location, can hardly be compared with those who live in densely populated regions - cities for example. Has any cognisance at all been taken of the mental effects of such increased isolation in the present crisis? Is it possible that the long term health effects of this forced isolation will have far more negative effects on the health of individuals than the virus itself… given that not everyone will get the virus and of those who do, many will survive. There is no doubt but that people who are vulnerable must be protected but is the present way the only good way?” Mary (F, 72 y)
4.2- Media portrayals of the pandemic and flows of (mis)information	Influences of mass media communications in participants’ affective states, as well as its role in shaping their knowledge about the virus and behaviors through the pandemic.	“The message has not always been 100% clear, but it has not anywhere. I suppose the main thing is to try and have clarity, make the message clear, and simple, so that everybody can understand it.” Darragh (M, 72 y)
4.3- Trust in experts, government, and institutions	Role of the perceived reliability, truth, or ability of experts, governments, and institutions to handle the pandemic effects.	“We have to rely on people like NPHET^a^ to make the right decisions for us as a community, and I accept what they are doing. You know, governments may have made some poor decisions, but COVID has been a learning exercise and I hope as we proceed on the COVID journey, we’ll learn from our mistakes and the community and government will learn from the mistakes.” Mark (M, 71 y)

a NPHET: National Public Health Emergency Team in Ireland.

Responses also indicate a wide range of uses of mass media communications, such as keeping informed about the pandemic spread and number of cases, as well as learning about best practices to minimize risk of infection or possible treatments, which contributed to “allowing people and empowering people to build up their common sense” (Barry, M, 78 y). However, participants such as Ruth (F, 66 y) reported that the heavy flow of information could “make it feel as if it’s never going to end” and lead to increased anxiety:

“It got to a stage that it was too much. I just needed to hear it once a day and then turn away from it. I just think there is a limit to seeing scenes in hospitals and listening to people who weren’t coping… Although I wouldn’t have missed it because I wanted to be in the loop, so I had to manage it.”

Strategies to manage media consumption included carefully selecting trusted sources and limiting the exposure times. Participants also highlighted the importance of clear language in public health communication and referred that transparency in the rationale for implementation of measures made them more inclined to comply and contributed to building trust.

### 3.5. Macro-system level (L5)

Definitions of macro-level determinants and quoted examples are provided in Table 6. Participants perceived that as a group, older people had been discriminated against because the general population considered COVID-19 a “disease of the old” and that public health restrictions resulted from the need “to protect people of certain ages that are so vulnerable to it, we must all adjust and live like this to protect you” (Geraldine, F, 68 y). According to participants, the portrayals of older people in public communications as a homogeneous and vulnerable group fed into previous socio-cultural stereotypes of older people as highly demanding of resources and low contributors to society. These negative perspectives deeply influenced how other people behaved around them and led to further age-discrimination and intergenerational divide:

**Table 6:** Details of socio-ecological determinants at the macro-system (L5).

Determinant	Definition	Illustrative quotes
5.1- Socio-cultural perspectives about aging and ageism	Perspectives about how the general society feels, thinks, and acts towards aging and older people.	“There is a perception out there that once one gets to 70 (the magic number), one is ill informed and/or too stupid to understand what is happening and is incapable of looking after oneself. It seems that older people are once again portrayed as objects or commodities who because of their age are a bit of a nuisance so the solution is to lock them up for the duration - cocoon, handy word but very disrespectful.” Louise (F, 72 y)
5.2- Socio-cultural expectations surrounding help seeking behaviors	Perspectives of how social norms influence help seeking behaviors and how these may be influenced by culture.	“Some people are proud, and they’ll refuse help. Like, you know, that crowd I was telling you about, they ring them up and say, ‘This number you can ring it any time, if you want to chat to someone, ring any time up to nine o’clock at night’. But they would not, because ‘Oh, I do not want to be disturbing people because there’s people more worse off than I am’, and they would be too proud to ask for help, and too proud to admit that there were lonely […] They will be saying, ‘Oh, I’m fine. I’m fine. I’m grand’, you know, and they put on a big smile, but they are not. I know they are not because I’ve been lonely myself at times.” Agnes (F, 70 y)
5.3- Discourse and social norms regarding mental health	Perspectives of the socio-cultural norms surrounding discourses about mental health and/or influencing the opportunity to discuss mental health issues.	“I’ll go forward, you know, doing the best I can, but I will not drink that kool-aid of toxic positivity that I get from other people. Ugh! You know, there’s nothing worse than the people with a great big smile, and oh, everything’s grand and, ‘oh, let us be positive and all of that’, you know? I can tell you what to do with your positivity.” Enda (NB, 66 y)
5.4- Global forces in an intrinsically connected world	Wider socio-cultural circumstances that transcend boarders and influence the course of the pandemic (i.e., rise of political extremes)	“This global pandemic that has exposed issues of racism, sexism, transphobia, xenophobia, you know, not just by Trump and his haters or the Brexiters^a^, there are plenty of them right here in Ireland […] That’s the far-right wing, finding an opportunity and exploiting it […] They’re organising, and strategizing, and rowing, you know, it’s sinister stuff.” Lorcan (M, 68 y)

a Brexiters: People in favor of the United Kingdom withdrawing from the European Union.

“In the very beginning almost every bulletin, every news, every announcement was about people catching COVID, and it was almost like they dismissed anybody of a certain age. In other words, they're going to die anyway. And in the very initial stages, I couldn't believe it, when we were actually put into a separate category, while they were speaking about the able-bodied person and prime person in their 30-40-50s. Then it was like, we were the cause of the pandemic spreading, in the sense that we were using up the hospital because the virus could kill us but wouldn't kill a young person. You know, created this divide.” Ciara (F, 66 y)

Anecdotes and media coverage of older people receiving sub-standard treatment because of their age, as well as the high number of cases and deaths in nursing homes, led to concerns that ageism could influence participants’ access to healthcare and the quality of services received in the case of a COVID-19 infection. For instance, Agnes (F, 70 y) recounted:

“Ten of the old people here in the small hospital got it then and died (…) I think that maybe if they weren't old, they might have been more conscientious about testing them, but because there were 80 or something, they said ‘oh, it should be alright’ but wasn’t.”

Direct and indirect age-discrimination experiences contributed to fears about becoming ill and frustration about societal responses; while discourses equating older age with declining capacities and low independence resulted in patronizing recommendations, which angered participants like Cathy (F, 73 y) who shared: “The over 70s were almost taken as if they were children again, I was very annoyed with that. I’m a thinking person, I certainly did not want to be told what to do.” Additionally, depictions of older people as dependent influenced participants’ help-seeking behaviors, as they feared losing their autonomy and dignity, as well as becoming a burden to others. For example, Bridget (F, 76 y), who was living alone and had formed a bubble with a couple in her neighborhood, reported that she had to carefully consider how often she could contact them to avoid impinging on their personal lives, even though she felt lonely and desired more social interactions. Similar responses also indicated a fear of asking for help because “others may need it more,” which highlights the benefits of community organizations and friends reaching out, as Thomas (M, 72 y) shared: “It felt very, very positive that people offered help without being asked. That made a big difference. It’s a lot better for someone to offer something than for you to have to ask them to do it. Feels better.”

Participants also had contrasting views about perceived cultural characteristics contributing to or hindering resilience. For instance, Niamh (F, 65 y) considered that “We have a habit of, particularly in Ireland, we love misery. Sometimes the people are whining a bit too much about little, small things,” while Odhran (NB, 66 y) reported “I dig deep for that Irish resilience, and the Irish sense of humor that I inherited from my Irish grandmother, who I never met, but I know I have it, and that’s sustaining.” Similarly, participants also held contrasting views about socially acceptable coping mechanisms and the opportunities to discuss their mental health. In this regard, Enda’s quote in Table 6 touches upon the implications of superficial solutions that may brush over more severe mental health challenges. Similarly, Noreen (F, 73 y) shared: “They go on and on about how you have to be stronger, even in at a time like this, and I do not want to be stronger. I’m fed up with it all. I mean, I want to put my feet up and eat cream cakes all day long (laughs),” which highlights the potential for some negative implications of social expectations regarding resilience.

### 3.6. Chrono-system level (L6)

The determinants identified at the chrono-system level relate to temporal and ephemeral determinants during the pandemic. Definitions and example quotes are provided in Table 7. Across participants’ narratives, the pandemic is described as an evolving event that is characterized by an abrupt beginning, followed by emerging knowledge about the virus, and several waves of increases in infections with a readjustment of public health measures. Accordingly, early stages of the pandemic are described as an uncertain period that is associated with contrasting positive and negative feelings with fear and anxiety on the one hand, and a sense of novelty and social solidarity on the other. As the pandemic unravelled through weeks and months, individual and communities put in place adaptive strategies and settled into new routines. In this regard Sarah (73 y, F) shared: “We were in on the drill and knew what the drill was: what we had to do, what we could do, what we were allowed to do, and we were all sticking through.” However, new waves of increasing number of infections paved the way for new stressors to emerge while communities support fizzled down. For instance, James (70 y, M) shared: “There’s a cumulative effect. I think the longer that it’s on, the more you feel you are really missing the kind of things that you could tolerate missing for a short while.” As such, public health advancements in treatment and prevention, particularly the COVID-19 vaccine, were viewed as a welcomed development that provided “some light at the end of the tunnel.”

**Table 7:** Detailed socio-ecological determinants at the chrono-system (L6).

Determinant	Definition	Illustrative quote
6.1- From managing the unknown to setting in a new normal	Related to perceptions of the pandemic as an unfolding event where it is possible to identify different stages that are associated with diverse emotional states.	“My confidence is much better now. I suppose we have grown accustomed to living with it. Initially, when the first lockdown came, there were no cars on the roads, people were scared to be travelling on a bus or travelling on a train. It used to be. I’d be very conscious of it. Even going to the dentist or going into the doctor surgery where you would be in close contact or going to the hairdresser. But now I’ve had the vaccine, I wear the mask, I’ve grown accustomed to this, we are living with it. That’s where we are at.” Paddy (M, 89 y)
6.2- Emotional fatigue and reducing support	Related to winding down of support and solidarity throughout the pandemic.	“I think at the beginning there was a rush of community groups reaching out. I think it probably has floundered a bit. Maybe there’s a fatigue in some of the organizations… I have not had any packages recently, that could be because of financial limitations. I’m not sure. But I would like to think that it’s not finished.” Greg (M, 72 y)
6.3- Other temporal determinants	Related to ephemeral characteristics of social and physical environments (i.e., weather, seasonal traditions).	“When January came and Christmas was over, people talked about the January blues. Weather-wise it was terrible, and it was awful looking out. There were so many evenings I thought ‘I do not remember getting dark as early as this before in the month of January’. But that’s maybe because I wasn’t sitting around hoping the day would last longer. I do not know, but I was very, very down in January.” Roisin (F, 70 y)

The fluctuation of affect and accumulation of stressors was also exacerbated by ephemeral conditions such as weather and seasonal traditions. Whereas darker, colder, and rainier months were associated with an increase in negative emotions; while warmer temperatures and more sunshine were associated with positive affect through more opportunities to take part in outdoor coping activities such as meeting with others at a safe-physical distance, walking or gardening.

## 4. Discussion

To our knowledge, this is the first study utilizing all the levels of Bronfenbrenner’s socio-ecological model to identify and categorize the many factors that have influenced the health and well-being of people aging-in-place during COVID-19. This exhaustive approach denotes the uniqueness of each individual experience but also highlights multi-scalar opportunities for interventions to support older people during public health crises by identifying junctions were short- and long-term vulnerability may emerge. According to results of this study, vulnerability is rarely linked to a single determinant and often emerges from multi-faceted interactions between individual and contextual circumstances that can be nested in the proximate, socio-cultural and/or policy environment. In the pandemic context, disruptions at several levels of everyday life had the potential to accelerate previous trajectories of vulnerability and even to become points of no-return but may have also presented new opportunities for personal growth if individuals were meet with appropriate resources and support.

As such, our findings reinforce that the wide arrange of pandemic experiences is reflective of the vast diversity of needs and capabilities among the older population (36). Moreover, in line with previous scholarship, findings suggest that a good person-environment fit, understood as a high degree of compatibility between individual’s needs and their opportunities to access suitable material, social and affective resources (37), may facilitate the timely development of adaptive strategies and successful coping mechanisms during a public health crisis. In contrast, poor person-fit environment and the unequal distribution of health enabling resources has the potential to stimulate or exacerbate poor health trajectories (4). This finding emphasizes the need to establish support services and physical environments that are crafted according to the very diverse needs and preferences of older individuals. Moreover, our results reinforce the notion that older people are not merely recipients of support but are active agents in their own health and well-being and may have a key role in supporting others (36, 38). Accordingly, catering for a heterogeneous older population should be integrated into support services at the community level and healthcare by closely collaborating with older people themselves (39).

These results also provide insights into the implications of one-size-fits-all approaches that lack recognition of the heterogeneity of older people. Echoing other COVID-19 studies (8), participants reported ambivalent outcomes related to the cocooning measure. While participants recognized it had provided protection from infection and that it had been necessary since the government was acting with a limited amount of evidence available and under time pressure, it overlooked unique circumstances among older people, which fueled ageist behaviors and social pressure to fit into a vulnerable identity. Previous evidence indicates that ageism may have strong influences on older people’s health and well-being by being internalized, which often leads to resentment towards others and affects individuals’ sense of agency and independence (40). Although blanket approaches may remain necessary in certain circumstances, counter measures to avoid unintended consequences include their implementation only for short periods of time, inbuilt pathways for ongoing adaptation and collaboration with the populations affected so it is feasible for policymakers to capture unintended effects in a timely manner and co-develop mitigation strategies. Additionally, results confirm that mass media communications have an important role in providing a clear message of the rationale of public health measures and in showcasing the heterogeneity of older people’s experiences, which can contribute to build intergenerational bonds (40, 41).

We acknowledge that the limitations of the present study include remote data collection, which may hinder communication between researchers and participants due to limited physical queues and technical difficulties, such as poor internet connection causing delays in online interviews. To compensate for these, the research team gave participants the opportunity to choose which method of data collection they preferred, and utilized active listening, prompts and verbal queues to build rapport. An additional strength is our multi-method approach to collect data from participants, which diminishes the risk of only capturing the experiences of older people who are comfortable with digital technologies. However, due to the limitations to meet face-to-face during the recruitment, we had to rely on remote strategies, such as contact with community organizations and older people representatives, as well as advertisements in public spaces (i.e., shops, pharmacies, places of worship, post offices) that may not have equal reach across Ireland. We suggest findings from this study should be expanded and triangulated with further studies focusing on different contexts or populations, as well as studies with complementary research methodologies, such as those utilizing longitudinal and/or nationally representative data.

## 5. Conclusion

Findings from this study present a snapshot of the experiences of people aging-in-place during a limited period of the pandemic. As indicated in the chrono-system, participants’ perspectives and needs are prone to change, which highlights individuals’ adaptive potential, as well as the potential fragility and resilience of our social and physical environments, and that of our community support and healthcare services. Resonating with the participant’s quote that illustrates individual resilience, the implication for public health practitioners and policy makers is to seek “to develop a normal way of being that allows us to survive challenges and difficulties.” Ultimately, our evidence indicates that developing pro-active and resilient interventions in non-emergency times may have the most potential for adaption during times of crisis, and that interventions seeking to support the aging population should place collaboration with older people at their core.

## Data availability statement

The datasets presented in this article are not readily available because of the sensitive nature of the data for this study. Requests to access the datasets should be directed to vivekaguzman@rcsi.ie.

## Ethics statement

The studies involving human participants were reviewed and approved by Royal College of Surgeons in Ireland Research Ethics Committee (REC202011028). The participants provided their written informed consent to participate in this study.

## Author contributions

VG, PC, NC, GH, AS, and PW: conceptualization and study design. VG: investigation, project administration, and writing—original draft preparation. VG, FD, RF, and MMP: methodology. VG, PC, NC, PW, and MP: analysis and interpretation. VG, FD, RF, PC, NC, PW, and MMP: writing—review and editing. FD, RF, and MMP: supervision. All authors contributed to the article and approved the submitted version.

## Funding

This research was funded by the Health Research Board (HRB) (grant SPHeRE-2019-1).

## Conflict of interest

The authors declare that the research was conducted in the absence of any commercial or financial relationships that could be construed as a potential conflict of interest.

## Publisher’s note

All claims expressed in this article are solely those of the authors and do not necessarily represent those of their affiliated organizations, or those of the publisher, the editors and the reviewers. Any product that may be evaluated in this article, or claim that may be made by its manufacturer, is not guaranteed or endorsed by the publisher.
